# Age-Related Differences in Surgical and Biochemical Outcomes Following Parathyroidectomy for Primary Hyperparathyroidism

**DOI:** 10.3390/jcm14217740

**Published:** 2025-10-31

**Authors:** Eun Jin Kim, Jin Kyong Kim, Sang-Wook Kang, Jandee Lee, Jong Ju Jeong, Kee-Hyun Nam, Woong Youn Chung

**Affiliations:** Department of Surgery, Yonsei University College of Medicine, Seoul 03722, Republic of Korea; thyrokim@yuhs.ac (E.J.K.);

**Keywords:** primary hyperparathyroidism, parathyroidectomy, older adults, bone turnover markers, surgical outcomes

## Abstract

**Background/Objectives**: Despite its increasing incidence in older patients, parathyroidectomy for primary hyperparathyroidism (PHPT) is frequently deferred owing to risks and age-related comorbidities and the limited evidence of age-specific surgical safety and biochemical outcomes. We evaluate age-related differences in clinical characteristics, perioperative outcomes, postoperative complications, and biochemical responses, including bone turnover markers, after parathyroidectomy for PHPT. **Methods**: We retrospectively enrolled 596 patients who underwent parathyroidectomy between 2009 and 2022, stratified into three age groups: <65, 65–74, and ≥75 years (Group A, *n* = 401; Group B, *n* = 141; and Group C, *n* = 54, respectively). Demographics, comorbidities, operative details, complications, pathology, and biochemical parameters were compared between the groups. **Results**: Older patients exhibited a higher prevalence of hypertension, cardiovascular disease, diabetes, osteoporosis, and chronic kidney disease (all *p* < 0.01), whereas multiple endocrine neoplasias were more frequent in younger patients (*p* = 0.002). Younger patients had a longer operation time (*p* = 0.006). There were no significant intergroup differences in postoperative hospital stay and complication rates, including transient hypoparathyroidism, hungry bone syndrome, and recurrent laryngeal nerve injury. Pathologic diagnoses were comparable, with single adenoma being most common (81.0–86.2%). The postoperative calcium and parathyroid hormone levels normalized in all groups. Younger patients had higher baseline bone turnover markers and demonstrated greater absolute reductions postoperatively (*p* = 0.030 and *p* = 0.042, respectively); however, improvements were observed in all age groups. **Conclusions**: When appropriately selected, parathyroidectomy is safe and effective in all age groups, including older patients with comorbidities. Considering its evident biochemical and skeletal benefits, age should not preclude surgical intervention for PHPT.

## 1. Introduction

Primary hyperparathyroidism (PHPT) is a prevalent endocrine disorder that is characterized by the autonomous hypersecretion of parathyroid hormone (PTH), which leads to hypercalcemia through increased bone resorption, augmented renal calcium reabsorption, and enhanced calcium absorption via elevated vitamin D activation [1,2]. Although historically considered a rare disease, the incidence of PHPT has markedly increased over the past few decades, particularly among postmenopausal women (female-to-male ratio 3:1) [3,4]. Most contemporary cases are asymptomatic or minimally symptomatic; however, untreated PHPT can cause complications, such as osteoporosis, nephrolithiasis, and neurocognitive and cardiovascular disturbances [5]. Surgical resection remains the only definitive treatment and effectively normalizes serum calcium and PTH levels and improves long-term outcomes [6,7]. However, a significant proportion of older patients continue to be underdiagnosed and undertreated [4,8]. The nonspecific symptoms of PHPT―such as fatigue, cognitive decline, and musculoskeletal pain―are frequently misattributed to aging, and delay both diagnosis and intervention [9]. Furthermore, concerns about surgical risks have contributed to hesitation in pursuing operative management in older adults [10,11]. However, there is increasing evidence that parathyroidectomy can be safely performed in older patients and confer outcomes comparable to those observed in younger cohorts [4,12]. Surgical intervention in this population improves bone health, reduces nephrolithiasis risk, and potentially enhances neurocognitive function [13,14]. Therefore, there is a growing consensus that age alone should not preclude surgical management of PHPT when indicated [15].

In this study, we aimed to evaluate the clinical characteristics, perioperative outcomes, biochemical responses, and complication rates after parathyroidectomy in older and younger patients undergoing PHPT. By providing contemporary real-world data, we intended to clarify the safety and benefits of surgical intervention across different age groups.

## 2. Materials and Methods

### 2.1. Study Design and Patient Selection

This retrospective cohort study involved a chart review of 596 patients who underwent parathyroidectomy for PHPT at Severance Hospital, Yonsei University Health System (Seoul, Republic of Korea) between February 2009 and December 2022. The diagnosis of PHPT was based on elevated serum calcium and intact PTH levels in conjunction with preoperative imaging studies. Patients were excluded if they had parathyroid carcinoma, lacked adequate laboratory or follow-up data, or were lost to follow-up. Additionally, patients with parathyroid cysts of uncertain pathology or necrotic findings were excluded (*n* = 4). This study was approved by the Institutional Review Board of Yonsei University (IRB No.4-2024-0682). Based on their age at the time of surgery, the participants were divided into three groups: Group A (<65 years), Group B (65–74 years), and Group C (≥75 years).

### 2.2. Preoperative Evlauation and Surgical Technique

All patients underwent preoperative localization studies, including high-resolution neck ultrasonography and at least one functional imaging modality such as technetium-99m sestaMIBI scintigraphy, parathyroid venous sampling, or 18-fluiricholine positron emission tomography/computed tomography (PET/FT). The choice of imaging modality was based on availability and physician discretion. All patients subsequently underwent open selective parathyroidectomy performed by experienced endocrine surgeons. When preoperative imaging provided clear localization, patients underwent focused parathyroidectomy.

Intraoperative parathyroid hormone (IOPTH) monitoring was introduced in May 2015 and was used in over 95% of cases performed thereafter (495 of 516 patients). PTH was measured 5 min after gland excision, and if the level did not decrease by 50% from the baseline, additional measurements were obtained at 10 and 15 min. Bilateral neck exploration was performed when preoperative studies filed to localized the lesion, multiglandular disease was suspected, or IOPTH failed to demonstrate a sufficient decline.

Intraoperative laryngeal neuromonitoring has been routinely applied since November 2018, with a usage rate exceeding 90% in subsequent cases (312 of 341 patients).

### 2.3. Data Collection and Outcomes

The data collected included patient demographics, underlying comorbidities (hypertension, coronary artery disease, diabetes mellitus, osteoporosis, nephrolithiasis, and chronic kidney disease), laboratory results, operative details, histopathological findings, and postoperative complications. Comorbidities were quantified using the Charlson–Deyo Comorbidity Index and preoperative surgical risk was assessed using the American Society of Anesthesiologists (ASA) physical status classification.

Biochemical parameters included serum calcium, intact PTH, 25-hydroxyvitamin D, albumin, and bone turnover makers, such as β-C-terminal telopeptide (β-CTx), and procollagen type 1 N-terminal propeptide (P1NP). Laboratory values were obtained immediately prior to surgery and at 6–12 months postoperatively. The mean follow-up duration was 965 days (range, 35–4759 days), and patients with a follow-up period shorter than 8 months were excluded from the bone turnover marker analysis.

Transient hypoparathyroidism was defined as a need for calcium supplementation that persisted beyond postoperative day 1 but resolved within 6 months. Permanent hypoparathyroidism was defined as requiring calcium supplementation beyond 6 months. Hunger bone syndrome was defined as sustained hypocalcemia requiring intravenous or high-dose oral calcium replacement despite the normalization of PTH levels. Recurrent laryngeal nerve injury was defined as persistent vocal fold immobility that was observed on postoperative laryngoscopic examination beyond 6 months after surgery or the need for definitive intervention, such as thyroplasty.

Board-certified endocrine pathologists confirmed the histopathological diagnosis. The pathological categories included single adenomas, double adenomas, and hyperplasia. All cases were independently reviewed by at least one pathologist who was blinded to the patient outcomes.

### 2.4. Statistical Analysis

Statistical analyses were performed using SPSS Statistics version 27.0 (IBM Corp., Armonk, NY, USA). Continuous variables were compared using one-way ANOVA or Kruskal–Wallis tests, and categorical variables were analyzed using the chi-square or Fisher’s exact tests. Trends across age groups were assessed using the Jonckheere–Terpstra test. Statistical significance was set at *p* < 0.05.

## 3. Results

Among the 596 patients who underwent parathyroidectomy for PHPT and were categorized into three age groups, the mean ages were 53.0, 69.3, and 78.3 years in Group A (<65 years, *n* = 401), Group B (65–74 years, *n* = 141), and Group C (≥75 years, *n* = 54), respectively. The female-to-male ratios were 294:107, 118:23, and 40:14, respectively (*p* = 0.044). The preoperative serum calcium and intact PTH levels were comparable across the groups (Table 1).

The prevalence of comorbidities increased with age (Table 2). The prevalence of hypertension, cardiovascular disease, diabetes, osteoporosis, and chronic kidney disease was significantly higher in the older age group (all *p* < 0.01). Multiple endocrine neoplasia (MEN) syndrome was more common in younger patients (7.5% in Group A vs. 0.7% in Group B, and 0% in Group C; *p* = 0.002). Nephrolithiasis and malignancy as a comorbidity showed no significant age-related differences (Table 2).

Surgical outcomes and postoperative complication rates were generally consistent across the age groups (Table 3). Bilateral parathyroid exploration was performed in 9.0%, 6.4%, and 9.3% of the patients in groups A, B, and C, respectively (*p* = 0.629). The operation time was significantly longer in Group A (*p* = 0.006) whereas the duration of hospital stay did not differ significantly (*p* = 0.095). Postoperative complications were infrequent and were not significantly associated with age. The rates of transient hypoparathyroidism, hungry bone syndrome, persistent hyperparathyroidism, transient hoarseness, and recurrent laryngeal nerve injury were all low and comparable across the groups (all *p* > 0.1).

The pathological diagnoses were similar across the age groups (Table 4). A single adenoma was the most common finding in all groups, and was observed in 86.2%, 83.0%, and 81.5% of patients in groups A, B, and C, respectively (*p* = 0.960). The mean tumor size and weight of single adenomas did not differ significantly between the groups. Double adenomas were rare and occurred in 3.0%, 2.1%, and 3.7% of patients in groups A, B, and C, respectively (*p* = 0.774). Hyperplasia was found in 11.5%, 14.9%, and 14.8% of patients in groups A, B, and C, respectively (*p* = 0.106), with comparable gland sizes and weights.

Postoperative serum calcium and PTH levels were normalized in all groups without significant differences (Table 2). Vitamin D levels tended to be higher in the older age groups (*p* = 0.023), whereas serum albumin levels showed a significant age-dependent decline (*p* < 0.001). Serum biochemical parameters and bone turnover markers were evaluated before and after parathyroidectomy to assess the surgical efficacy (Table 5). Preoperatively, Group A showed higher β-CTx and P1NP compared with Group B and C (both *p* = 0.001). Postoperatively, both markers decreased significantly in all age groups (β-CTx *p* = 0.002; P1NP *p* = 0.029) and P1NP levels decreased significantly across all age groups (*p* = 0.002 and *p* = 0.029, respectively), with greater absolute reductions in younger patients (Δβ-CTx −0.555 ± 0.55 vs. −0.397 ± 0.53 and −0.425 ± 0.40 ng/mL, *p* = 0.030; ΔP1NP −41.41 ± 83.6 vs. −21.35 ± 37.8 and −16.62 ± 38.5 ng/mL, *p* = 0.042).

## 4. Discussion

PHPT is a common endocrine disorder that is primarily caused by a single parathyroid adenoma, with less frequent etiologies including glandular hyperplasia or double adenoma (2–5%) [16]. Furthermore, PHPT may arise in hereditary syndromes such as MEN, familial hypocalciuric hypercalcemia, and hyperparathyroidism-jaw tumor syndrome [1]. Excessive PTH secretion promotes osteoclastic bone resorption, leading to chronic hypercalcemia, which in turn results in osteopenia or osteoporosis, and contributes to complications, such as nephrolithiasis, neurocognitive impairment, gastrointestinal symptoms, and cardiovascular morbidities [5].

PHPT is particularly prevalent in older individuals, with epidemiologic studies reporting that as many as 1 in 100 women of African descent and 1 in 300 Asian women older than 70 years are affected [17]. Although the prevalence of the disease is substantial among the older population, it is frequently under-recognized and under-diagnosed in this age group. This underestimation is partly due to the nonspecific, multi-systemic nature of PHPT symptoms, which may be attributed to aging or coexisting comorbidities [9,18]. However, advancements in routine biochemical screening have improved diagnostic rates, allowing for more timely identification of asymptomatic or minimally symptomatic cases [3,4].

Surgical resection of the overactive parathyroid gland(s) remains the only definitive treatment for PHPT because it directly addresses the source of hormone excess and provides durable curative outcomes across a broad range of clinical presentations [19]. Although pharmacological agents, such as calcimimetics, can alleviate some physiological consequences of hypercalcemia, they do not reverse the underlying disease process [20,21]. Moreover, long-term cost-effectiveness is inferior to surgical management [3]. Especially in older patients, surgery offers substantial benefits, such as normalization of serum calcium and PTH levels, reduced risk of life-threatening complications such as fractures or cardiovascular events, and potential improvements in the overall quality of life [8,22,23].

In our cohort analysis, the oldest patients (Group C) showed surgical outcomes comparable to those of younger patients (groups A and B), with no significant increase in postoperative complications or length of hospital stay. Interestingly, the operative time was longer in younger patients, likely due to a higher rate of bilateral neck exploration―even in cases without bilateral resection―or additional procedures, rather than age-related factors. These findings align with those of recent studies, suggesting that parathyroidectomy is well tolerated in older adults when performed in experienced centers [3,24].

Although older patients frequently present with higher ASA physical status scores, our data indicate that surgery was not withheld in patients without markedly elevated ASA scores. Notably, none of the older patients with ASA ≥ 3 underwent surgery, reflecting a possible bias toward conservation management in high-risk individuals. However, our findings support the notion that when appropriately selected, older patients derive significant clinical benefits from surgery without incurring excessive operative risks [14,25].

This study revealed age-related differences in the postoperative biochemical responses and bone turnover following parathyroidectomy for PHPT. Postoperative calcium and PTH levels normalized uniformly across the age groups, which supports the effectiveness of surgery throughout the age spectrum. However, serum albumin levels decline with advancing age, possibly reflecting age-associated changes in nutritional or physiological status. Postoperative vitamin D levels tended to be higher in the older age groups, although the difference was not statistically significant. This may be attributed to the more aggressive vitamin D supplementation in older patients, either orally or intramuscularly, both in terms of dosage and frequency, during the perioperative period. Such practices likely reflect clinicians’ awareness of baseline vitamin D insufficiency and greater fracture risk in older individuals [6,26].

Regarding bone turnover, younger patients demonstrated higher baseline levels of β-CTx and P1NP and a more pronounced reduction in these markers following parathyroidectomy. This suggests that bone remodeling is more dynamic in younger individuals and may respond more robustly to the resolution of hyperparathyroidism [27,28]. Nonetheless, older patients exhibited meaningful improvements in bone turnover markers. These results underscore the physiological benefits of parathyroidectomy regardless of age. Consistent with previous studies, parathyroidectomy remains a safe and effective treatment, even in older adults with comorbidities, provided they are appropriately selected [12,15,25,29].

This study has several limitations. First, it was conducted retrospectively at a single tertiary referral center with experienced endocrine surgeons, which may limit the generalizability of the findings to other institutions with varying surgical volumes and practices. Second, although the sample size was relatively large, the proportion of patients aged ≥75 years was comparatively small, potentially limiting the statistical power to detect age-specific differences in outcomes, especially rare complications. Third, we did not evaluate patient-reported outcomes such as quality of life, neurocognitive function, or frailty status, which are clinically relevant, especially in older adults undergoing parathyroidectomy. Finally, potential confounders such as bone mineral density, fracture history, nutritional status, and pharmacologic treatment (e.g., biosphosphonates or vitamin D analogs) were not uniformly available for adjustment. These factors should be considered when interpreting the result, and prospective, multicenter studies with longer follow-up are warranted.

## 5. Conclusions

In conclusion, age alone should not constitute a contraindication for parathyroidectomy in patients with PHPT. Careful perioperative assessment and appropriate patient selection facilitate safe and effective surgical outcomes, even in older individuals. Given the risk of fractures, renal and cardiovascular complications, and diminished quality of life associated with untreated PHPT, a more aggressive surgical approach may be warranted for older patients who are otherwise suitable candidates for surgery.

## Figures and Tables

**Table 1:** Age group-stratified baseline clinical characteristics of patients.

Variables	Group A (<65 Years) (*n* = 401)	Group B (65–74 Years) (*n* = 141)	Group C (≥75 Years) (*n* = 54)	*p*
Clinical Characteristics				
Age (years)	49.58 ± 11.4 (19–64)	68.98 ± 2.95 (65–74)	78.44 ± 3.44 (75–87)	
Sex (female/male, (female %))	294/107 (73.3%)	118/23 (83.7%)	40/14 (74.1%)	0.044 *
Preoperative Serology				
Calcium (mg/dL)	11.17 ± 1.00 (8.90–17.00)	11.20 ± 1.17 (8.00–16.70)	11.16 ± 0.89 (9.20–13.40)	0.927
PTH (pg/mL)	164.1± 120.3 (40.9–831.0)	159.9 ± 120.1 (49.0–700.0)	158.4 ± 100.1 (42.3–656.0)	0.778
25(OH)D (ng/mL)	18.74 ± 9.42 (3.46–56.71)	22.14 ± 12.44 (3.50–99.56)	20.97 ± 12.84 (2.89–67.89)	0.004 *
Albumin (g/dL)	4.53 ± 0.33 (3.10–5.40)	4.38 ± 0.35 (3.20–5.00)	4.12 ± 0.51 (2.70–4.90)	0.000 *
Postoperative Serology				
Calcium (mg/dL)	9.04 ± 0.36 (8.2–10.5)	9.00 ± 0.36 (7.7–10.0)	9.01 ± 0.36 (8.5–10.0)	0.254
PTH (pg/mL)	15.43 ± 11.81 (3.5–127)	16.07 ± 12.14 (2.7–112)	18.93 ± 15.6 (6.4–99.9)	0.018 *
25(OH)D (ng/mL)	29.16 ± 10.12 (4.89–68.48)	31.13 ± 10.18 (9.67–72.0)	31.99 ± 12.42 (8.21–73.52)	0.023 *
Albumin (g/dL)	4.51 ± 0.33 (3.1–5.4)	4.37 ± 0.38 (2.6–5.0)	4.12 ± 0.51 (2.7–4.9)	<0.001 *

Data are expressed as number (%) or mean ± SD (range). * Statistical significance set at *p* < 0.05. Abbreviations: PTH, parathyroid hormone; 25(OH)D, 25-hydroxyvitamin D.

**Table 2:** Age group-stratified comorbidities and the ASA score.

	Group A	Group B	Group C	*p*
Comorbidities (*n* (%))	276 (68.8%)	126 (89.3%)	51 (94.4%)	<0.001 *
HTN	122 (30.4%)	80 (56.7%)	49 (90.7%)	<0.001 *
CAOD	7 (1.7%)	21 (14.8%)	11 (20.4%)	<0.001 *
DM	63 (15.7%)	29 (20.5%)	19 (35.2%)	0.002 *
CKD	12 (3.0%)	18 (12.7%)	9 (16.7%)	<0.001 *
Nephrolithiasis	107 (25.9%)	41 (29.0%)	14 (25.9%)	0.815
Comorbid malignancy	27 (6.7%)	12 (8.5%)	7 (13.0%)	0.240
Osteoporosis	227 (56.6%)	121 (85.8)	49 (90.7%)	<0.001 *
MEN	29 (7.5%)	1 (0.7%)	0 (0%)	0.002 *
ASA Score, *n* (%)				<0.001 *
1	76 (19.0%)	4 (2.8%)	0 (0%)	
2	246 (61.3%)	70 (49.6%)	5 (9.3%)	
3	80 (20.0%)	65 (46.1%)	49 (90.7%)	
4	3 (0.7%)	2 (1.4%)	0 (0%)	

Data are expressed as number (%). * Statistical significance set at *p* < 0.05. Abbreviations: HTN, hypertension; CAOD, coronary artery occlusive disease; DM, diabetes mellitus; CKD, chronic kidney disease; MEN, multiple endocrine neoplasia; ASA, American Society of Anesthesiologists physical status classification system.

**Table 3:** Surgical outcomes and postoperative complications staratified by the age groups.

	Group A	Group B	Group C	*p*
Surgical outcomes				
Bilateral operation	36 (9.0%)	9 (6.4%)	5 (9.3%)	0.629
Operation time (minutes)	53.79 ± 20.91 (23–145)	46.42 ±19.21 (20–164)	48.04 ± 16.39 (27–97)	0.006 *
Postoperative hospital stays (days)	2.72 0.81 (1–10)	2.85 ± 1.28 (2–16)	2.98 ± 0.54 (2–5)	0.095
Postoperative complications				
Transient hypoparathyroidism	62 (15.5%)	21 (14.9%)	5 (9.3%)	0.483
Hunger bone syndrome	12 (3.0%)	3 (2.1%)	0 (0%)	0.539
Persistent hyperparathyroidism	2 (0.5%)	1 (0.7%)	1 (1.9%)	0.355
Recurrent laryngeal nerve injury	0 (0%)	1 (0.7%)	0 (0%)	0.327

Data are expressed as number (%) or mean ± SD (range). * Statistical significance set at *p* < 0.05.

**Table 4:** Pathological features of parathyroid specimen stratified by the age groups.

	Group A	Group B	Group C	*p*
Single adenoma	346 (86.2%)	117 (83.0%)	44 (81.5%)	0.960
Size (cm)	1.49 ± 0.77 (0.3–5.6)	1.48 ± 0.7 (0.4–3.8)	1.43 ± 0.63 (0.5–3.2)	0.904
Weight (mg)	0.73 ± 1.03 (0.1–10.8)	0.65 ± 0.77 (0.1–3.1)	0.60 ± 0.44 (0.1–2.0)	0.711
Double adenoma	13 (3.0%)	3 (2.1%)	2 (3.7%)	0.774
Size (cm)	1.62 ± 0.57 (0.8–2.5)	1.07 ± 0.49 (0.5–1.4)	1.50 ± 0.71 (1.0–2.0)	0.351
Weight (mg)	0.79 ± 0.94	–	–	
Hyperplasia	46 (11.5%)	21 (14.9%)	8 (14.8%)	0.106
Size (cm)	1.31 ± 0.60 (0.3–3.0)	1.56 ± 0.88 (0.4–4.4)	1.24 ± 0.41 (0.4–1.7)	0.240
Weight (mg)	0.41 ± 0.45 (0.1–1.7)	0.36 ± 0.16 (0.1–0.6)	0.13 ± 0.58 (0.1–0.2)	0.499

Data are expressed as number (%) or mean ± SD (range). Statistical significance set at *p* < 0.05.

**Table 5:** Changes of bone turnover markers stratified by the age groups.

	Group A	Group B	Group C	*p*
Preoperative				
β-CTx (ng/mL)	0.882 ± 0.64 (0.024–4.26)	0.640 ± 0.58 (0.031–3.85)	0.721 ± 0.52 (0.056–2.23)	0.001 *
P1NP (ng/mL)	93.04 ± 109 (10.0–969)	57.36 ± 42.9 (7.90–232)	57.55 ± 34.4 (13.1–182)	0.001 *
Postoperative (3–6 months)				
β-CTx (ng/mL)	0.340 ± 0.27 (0.038–2.05)	0.241 ± 0.15 (0.027–1.71)	0.291 ± 0.24 (0.058–1.14)	0.002 *
P1NP (ng/mL)	49.58 ± 42.0 (11.9–511)	36.68 ± 19.4 (7.40–118)	41.62 ± 34.4 (11.1–192)	0.029 *
Pre- to postoperative Change				
β-CTx (ng/mL)	−0.555 ± 0.55	−0.397 ± 0.53	−0.425 ± 0.40	0.030 *
P1NP (ng/mL)	−41.41 ± 83.6	−21.35 ± 37.8	−16.62 ± 38.5	0.042 *

Data are expressed as number (%) or mean ± SD (range). * Statistical significance set at *p* < 0.05. Abbreviations: β-CTx, β-C-terminal telopeptide of type I collagen; P1NP, procollagen tyle 1 N-terminal propeptide.

## Data Availability

The datasets generated and/or analyzed during the current study are not publicly available due to privacy restrictions but are available from the corresponding author on reasonable request.

## Abbreviations

The following abbreviations are used in this manuscript:

PHPT	Primary hyperparathyroidism
PTH	Parathyroid hormone
ASA	American Society of Anesthesiologists (physical status classification)
β-CTx	β-C-terminal telopeptide
P1NP	Procollagen type 1 N-terminal propeptide
